# Immunoexpression of PD-L1, CD4+ and CD8+ cell infiltrates and tumor-infiltrating lymphocytes (TILs) in the microenvironment of actinic cheilitis and lower lip squamous cell carcinoma

**DOI:** 10.1590/1678-7757-2021-0344

**Published:** 2022-02-21

**Authors:** Vinícius Gonçalves de Souza, Damilys Joelly Souza Santos, Ana Gabriela Silva, Rosy Iara Maciel de Azambuja Ribeiro, Adriano Mota Loyola, Sérgio Vitorino Cardoso, Carla Silva Siqueira Miranda, Ludimila Paula Vaz Cardoso

**Affiliations:** 1 Universidade Federal de Jataí Jataí Goiás Brasil Universidade Federal de Jataí, Curso de Medicina, Jataí, Goiás, Brasil.; 2 Universidade Federal de Jataí Jataí Goiás Brasil Universidade Federal de Jataí, Ambulatório de Diagnóstico Estomatológico do Sudoeste Goiano (ADESGO), Jataí, Goiás, Brasil.; 3 Universidade Federal de São João del-Rei Minas Gerais Brasil Universidade Federal de São João del-Rei, Curso de Medicina, Laboratório de Patologia Experimental, Minas Gerais, Brasil.; 4 Universidade Federal de Uberlândia Faculdade de Odontologia Uberlândia Minas Gerais Brasil Universidade Federal de Uberlândia, Faculdade de Odontologia, Patologia Oral, Uberlândia, Minas Gerais, Brasil.

**Keywords:** Cheilitis, Immunotherapy, Squamous cell carcinoma of head and neck, B7-H1 antigen, Tumor-infiltrating lymphocytes

## Abstract

Lower lip squamous cell carcinomas (LLSCC) could be associated with a previous history of potentially malignant oral diseases (PMOD), especially actinic cheilitis (AC), with high sun exposure being a well-described risk factor. Immune evasion mechanisms, such as the PD-1/PD-L1 (programmed cell death protein 1/programmed death-ligand 1) pathway has been gaining prominence since immunotherapy with immune checkpoint inhibitors showed a positive effect on the survival of patients with different types of neoplasms. Concomitant with the characterization of the tumor microenvironment, the expression of either or both PD-1 and PD-L1 molecules may estimate mutual relations of progression or regression of the carcinoma and prognostic values of the patient.

**Objective:**

Considering the importance of tumor microenvironment characterization, this study aims to determine the immunoexpression of PD-L1 and correlate with the frequency of CD4+ and CD8+ cells in AC and LLSCC lesions and with tumor-infiltrating lymphocytes (TILs) in LLSCC and its relationship with histopathological characteristics.

**Methodology:**

This sample includes 33 cases of AC and 17 cases of LLSCC. The cases were submitted to histopathological analysis and to CD4+, CD8+, and PD-L1+ cell determination by immunohistochemistry.

**Results:**

There was a significant difference among the frequencies of CD4+, CD8+, and PD-L1+ cells between AC and LSCC cases, higher in the last group. Moreover, histopathological and atypical changes in AC and LLSCC were correlated with the frequencies of PD-L1+, CD4+, and CD8+ cells. In AC, PD-L1+ cases had a low frequency of CD4+ cells, but on the other hand, PD-L1+ cases of LLSCC had a higher frequency of CD4+ and CD8+ cells.

**Conclusion:**

Therefore, the PD-L1 molecule may be a potential escape route for the immune response in oral lesions, but the mechanisms differ between AC and LLSCC. Future studies related to immune evasion and immunotherapy in oral lesions should consider the analysis of inflammatory infiltrate and TILs.

## Introduction

Actinic cheilitis (AC) is characterized by a chronic inflammatory lesion that preferentially affects light-skinned individuals who are constantly exposed to solar radiation.^1^ It is considered a potentially malignant oral disorder (PMOD) and its histopathological characterization is based on the findings of superficial inflammatory infiltrate and dysplastic changes of varying degrees in the epithelium, which may determine the potential for the development of lower lip squamous cell carcinoma (LLSCC).^2^ Additionally, the presence of solar elastosis is also noted, considered a marker of prolonged chronic sun exposure.^3^

The LLSCC is usually asymptomatic in the early stages. The clinical features are similar to AC and might include: white or erythematous atrophic plaques, fissures, and crusts. The lesions progress to a persistent, well-delimited, and infiltrative ulcer. Bleeding, nodularity, crusts, exophytic mass, and cervical lymphadenopathy could also occur. Most of the cases show a slow disease progression, and the lesion location may facilitate the diagnosis. However, some cases could progress with local invasion and metastasis that lead to a worse prognosis. Histologically, it is possible to observe cell and nuclear atypia associated with intense inflammatory conditions, focal areas of necrosis, and varying degrees of tumor infiltration.^3^ The histopathological classification of malignancy proposed by the World Health Organization (WHO) relies on the degree of differentiation or anaplasia of cells. The WHO grouped these neoplasms into the following groups: well-differentiated, moderately-differentiated, and poorly-differentiated.^4^

PMOD and carcinomas are usually recognized by the immune system which induces an inflammatory response, contributing to the regression of the lesion and preventing progression to neoplasms.^5^ On the other hand, the inflammatory profile, in some cases, may show pro-tumor activity, favoring PMOD and neoplasms to progress.^6^

The evasion mechanisms to the immune response are fundamental for treatment and prognosis, along with the pattern of the inflammatory microenvironment of these types of diseases. The various altered neoantigens expressed in PMOD and cancer can adopt escape mechanisms to inhibit the immune response, such as PD-1/PD-L1 (programmed cell death protein 1/programmed death-ligand 1) interaction, maintaining a disorderly progression.^7,8^

The PD-1 molecule exerts an inhibitory effect on several cell types – such as T cells, NK cells, B cells, monocytes, dendritic cells, and macrophages – modulating cytokine production and cell proliferation. The PD-L1, ligand of PD-1, is expressed in dendritic cells, macrophages, T cells, B cells, and neoplastic cells, so its expression is dependent on the tissue microenvironment and cytokines.^9,10^ The interaction between PD-1 and PD-L1 offers inhibitory signals, making the T cell anergic to its antigenic targets.^10,11^

In this context, the literature shows that the response to conventional cancer treatment is worse when malignant lesions present the expression of these molecules,^9^ since the PD-1/PD-L1 interaction pathway protects the tumor against attacks, mediated by the immune response, and increases the possibility of malignancy. Many clinical trials have used monoclonal antibodies, known as immune checkpoint inhibitors, to block this pathway and increase the chances of having an adaptive immune response in treated patients.^12-14^ Therefore, it is necessary to estimate the frequency of immune expression of these molecules in tumor tissues to select patients eligible for this type of immunotherapy.

In addition to determining the PD-L1, the inflammatory infiltrate – whether perilesional or stromal of dysplastic and neoplastic lesions – is essential to set the immune response against the altered antigens. Previous studies show that the density and distribution of tumor-infiltrating lymphocytes (TILs) have a significant impact on the prognosis of patients with various types of carcinoma.^14-16^ Furthermore, the antitumor immune response is strongly mediated by TILs, especially by CD8^+^ cytotoxic T lymphocytes.^11,17^

So far, several studies have been developed characterizing the role of PD-1 and PD-L1 molecules as biomarkers of therapeutic response and prognosis. They consider the employment of immune checkpoint inhibitors in head and neck cancer, such as pembrolizumab and nivolumab, both approved by the Food and Drug Administration (FDA).^18-20^ However, in oral cancer, the role of other components of the tumor microenvironment, such as inflammatory infiltrate, TILs, and CD4^+^ and CD8^+^ expression, remains to be better explored. Hence, it is essential to characterize the microenvironment in these disorders, understand their immunological profile, and analyze the influence of clinical and histopathological factors, as well as their association with the progression of the disease.

Thus, this study aims to determine the PD-L1 immunoexpression and to correlate the frequency of CD4^+^ and CD8^+^ cells in AC and LLSCC lesions and TILs in LLSCC lesions and their relationship with clinical and histopathological characteristics.

## Methodology

### Selection of tissue sample

A total of 50 tissue samples of AC (n=33) and LLSCC (n=17) from biopsies or surgical resections were selected from different sources. An anatomopathological report was issued from the analysis of these samples, which was performed by pathologists with experience in the area.

Inclusion criteria for tissue samples included consulting the database of these laboratories to identify cases in which the conclusion of the histopathological analysis was of AC or LLSCC. After selecting the cases, the respective anatomopathological examination request forms and medical and dental records were consulted to obtain sociodemographic data of the patients (age, sex, and ethnicity) and clinical data of the lesions (size, color, nature, etiology, signs, symptoms, diagnostic hypothesis, treatment, histological grade, and clinical stage of LLSCC).

The present study has the approval of the research ethics committees (protocol #3,443,761; #844,944). All of these materials were stored properly as paraffin-embedded tissue fragments in a biorepository. For this reason, this study was exempted from applying the Informed Consent Form.

### Tissue sample cutting and microscopic analysis

The selected paraffin-embedded materials were cut by microtome sections (Leica RM2245 Wetzlar Microtome) to obtain blocks with consecutive cuts of 4 μm thick. Five serial tissue cuts of AC and LLSCC were performed: one for routine staining, three for immunohistochemical reactions, and one for the control case. The slides were stored in a freezer (-20ºC) until the next steps.

The slide for routine staining was subjected to the Hematoxylin and Eosin (H&E) method^21^ to be reassessed by two experienced pathologists, who selected only those cases that presented the typical diagnostic characteristics of each of the two diseases.

Thus, to define the AC cases, the parameters used were: localized lip lesion; inflammatory infiltrate pattern and profile (mild, moderate, or intense infiltrate); solar elastosis, according to the amount of involvement, radial and vertical (mild, moderate or intense);^22^ histological gradation of dysplasia, with minimally mild dysplasia of the epithelium lining^4^; and quantity of mitosis, analyzed in 10 fields with 400x magnification, according to the criteria of Elston and Ellis.^23^

The definitions of the LLSCC cases were based on the following parameters: pattern and inflammatory profile being classified as mild, moderate, or intense infiltrate, regardless of whether the infiltrate is stromal or not; degree of tumor differentiation, such as well, moderately, and poorly^4^; quantity of mitoses in the field of cell infiltration, analyzed in a 400x increase. The analysis of TILs was based on a modified version of the protocols described in the “International TILs Working Group 2014”^24^ and the “VENTANA PD-L1 protocol (SP142) Assay for Triple Negative Breast Carcinoma”, focusing on the inflammatory infiltrate present in the stroma of the lesion. Briefly, the steps of evaluation included:

- Cells located in the stromal compartment of the tumors or peritumoral areas, bordering the lesion, were analyzed;
- Any Inflammatory infiltrates in areas distant from the stroma or the tumor margin were excluded from the analysis;
- The TILs were considered to be any type of leukocytes that were in the area mentioned above, including mononuclear and polymorphonuclear, as long as they were not considered cell debris, nor were in areas of necrosis or artifacts;
- The evaluated cuts had a thickness of 4 μm and were analyzed at 200x magnification, in a panoramic way, without considering the hot spot, so as not to mask the real amount of inflammatory infiltrate throughout the tumor stroma;
- Analyses were performed independently by two pathologists with knowledge in the area as stated previously. Discrepant cases were reviewed and reclassified;
- The quantitative analysis of TILs ranges from 0% to 90%. Cases then were subcategorized as TILs^low^ – 0% to 40% and TILs^high^ – 41% to 90%.

### Immunohistochemistry reaction for CD4, CD8, and PD-L1

Tissue samples containing 4 μm thick sections on silanized slides were subjected to immunohistochemistry reaction to individually determine the expression of CD4, CD8, and PD-L1 molecules.

After deparaffinization and hydration of tissue cuts in baths with decreasing concentrations of ethyl alcohol, the slides were incubated at room temperature with ammonium hydroxide 24% - 26% in 95% ethyl alcohol, for 10 min, to remove the formic pigment. After being washed with distilled water, antigenic recovery for CD4/CD8 and PD-L1 determinations was performed by incubation with citrate buffer (DINÂMICA^®^) pH 6.0, heated to a temperature of 100ºC for 20 min for CD4/CD8 and at 121 ºC for PD-L1. Blocking of endogenous peroxidase was performed in a 3% hydrogen peroxide solution (DINÂMICA^®^) for 40 min (for CD4/CD8) and 20 min (for PD-L1) at room temperature and protected from light. The slides containing the primary antibodies anti-CD4 (clone H-370, 1: 300, Santa Cruz Biotechnology^®^), anti-CD8 (clone C8/144B, 1: 300, DAKO^®^), and anti-PD-L1 (clone CAL10, 1:400, Biocare Medical^®^) were incubated for 18-24 h (overnight) at 4-8ºC in a humid chamber. The developer system used for CD4 and CD8 immunohistochemistry was the LSK2 System-HRP kit (K0675) from DAKO^®^. For determining PD-L1, the Starr Trek Universal HRP Detection System kit (Biocare Medical^®^) was used. Negative controls for all reactions were incubated with Tris-buffered saline (TBS).

All slides submitted to immunohistochemistry reactions were counterstained with Meyer’s hematoxylin for 30 s at room temperature and dehydrated in baths with increasing concentrations of ethyl alcohol. Subsequently, the slides were immerged in xylene and assembled with a non-aqueous resin solution (Canada Balsam).

The positive controls of all immunohistochemistry reactions were constituted by tissue samples of tonsils, the methodological conditions of which were the same as the cases. The negative controls consisted of tissue samples from the respective cases.

### Immunohistochemistry analysis for CD4, CD8, and PD-L1

The slides were scanned to obtain the images using the Aperio AT2 Scanner Slides (Leica Biosystems Imaging) and the images were obtained in 200x magnification using the ImageScope system (Leica Biosystems Imaging).

The analysis of CD4^+^ and CD8^+^ cells was performed by scanning the entire slide in AC cases and LLSCC cases, only in the region of tumoral and peritumoral stroma, using the same methodology described for TILs above.

The analysis of the PD-L1 immunoexpression followed strict and coordinated criteria based on this the following sequence:

- Before immunohistochemistry reactions, all H&E slides were analyzed for tissue integrity and viability;
- The positive control slides (tissue samples from tonsils) of the reactions in immunohistochemistry were analyzed to verify their positivity;
- The negative control slides (slides-case) were analyzed to check for negative cases;
- The slides-case of the immunohistochemistry reactions were analyzed concurrently with the negative control slides and the H&E slides to certify the specificity of immunohistochemistry marking and elimination of any type of artifact.

The determination of the PD-L1 positive (PD-L1^+^) cells and the expression profile (membrane or cytoplasmic) followed recommendations of the consensus by the Society for Immunotherapy of Cancer for the Treatment of Squamous Cell Carcinoma of the Head and Neck (HNSCC). It considered a positive expression to be greater than or equal (≥) to 50% in tumor cells (tumor proportion score – TPS) or ≥ 1% for combined staining of tumor cells and immune cells – IC (combined positive score – CPS).^12^ However, for this study, which includes lip carcinoma, cases that showed expression only in IC were also evaluated. For LLSCC, peritumoral tissues were not considered for analysis. The stratification of PD-L1 immunoexpression was performed according to the percentage of tissue marking, found in greater quantity in the lesions. This stratification was defined as 1%, between 1% and 5%, between 5% and 20%, and > 20%.

### Statistical analysis

Statistical analysis was performed using SPSS Version 22.0.0.0 statistical software package. By considering the number of cases included and the distribution of data, non-parametric statistical tests were used in this study. The correlation between the immunoexpression of PD-L1, CD4, and CD8 molecules and the histopathological variables was performed using Spearman’s correlation test. In addition, the chi-square association test (χ^²^) was used for the evaluation of immunohistopathological microenvironments and the immunoexpression of the molecules mentioned in the two lesions under study. The level of significance adopted for all analyses was p<0.05.

## Results

### Epidemiological and histopathological characterization of AC and LLSCC cases

The epidemiological characteristics of the individuals and the histopathological classification of tissue lesions of AC and LLSCC selected for this study are shown in Table 1. The AC group was composed of 78.8% of male individuals, with a median age of 50 years (range 34-80 years), 51.5% of white ethnicity, and history of chronic exposure to ultraviolet (UV) radiation and agricultural workers in 15.2%. Additionally, 18.2% and 9.1% of the individuals reported tobacco and alcohol use, respectively. The LSCC group included 88.2% of male individuals, with a median age of 56 years (range 38-87 years), 52.9% of white ethnicity, and 11.8% of agricultural workers. Furthermore, 52.9% and 41.2% reported tobacco and alcohol use, respectively.

**Table 1:** Epidemiological characteristics and histopathological classification of AC and LLSCC cases

Variables		AC (n=33) n(%)	LSCC (n=17) n (%)	p	χ^²^
Gender				0.410	0.678
	Male	26 (78.8)	15 (88.2)	-	-
	Female	07 (21.2)	02 (11.8)	-	-
Age group (years old)				-	-
	Median (interval) ^a^	50 (34-80)	56 (38-87)	-	-
Ethnicity ^b^				0.329	2.224
	White	17 (51.5)	09 (52.9)	-	-
	Brown	06 (18.2)	02 (11.8)	-	-
Professional occupation ^c^				0.732	0,117
	Rural worker	05 (15.2)	02 (11.8)	-	-
	Others	07 (21.2)	04 (23.5)	-	-
Smoking status ^d^		06 (18.2)	09 (52.9)	0.266	1.236
Alcohol user ^e^		03 (9.1)	07 (41.2)	0.889	0.019
Solar elastosis				-	-
	Mild	05 (15.2)	NA	-	-
	Moderade	10 (30.3)	NA	-	-
	Intense	18 (54.5)	NA	-	-
Inflammatory infiltrate				0.042	3.766
	Mild	19 (57.6)	05 (29.4)	-	
	Moderade	07 (21.2)	05 (29.4)	-	
	Intense	07 (21.2)	07 (41.2)	-	
Dysplasia				-	-
	Mild	18 (54.6)	NA	-	-
	Moderade	11 (33.3)	NA	-	-
	Intense	04 (12.1)	NA	-	-
Presence of mitosis		16 (48.5)	09 (52.9)	0.765	0.089
Gradation				-	-
	WD	NA	09 (52.9)	-	-
	MD	NA	05 (29.4)	-	-
	PD	NA	03 (17.6)	-	-
TILs				-	-
	0% - 40%	NA	09 (52.9)	-	-
	41% - 90%	NA	08 (47.1)	-	-
PD-L1 expression				0.046	3.822
.....0%		27 (81.8)	11 (64.7)	-	-
.....1%		04 (12.1)	03 (17.6)	-	-
.....1% - 5%		02 (6.1)	02 (11.8)	-	-
	5% - 20%	00 (0.0)	01 (5.9)	-	-
CD4+ cells frequency				0.029	4.741
	Low	24 (72.7)	7 (41.2)	-	-
	High	09 (27.3)	10 (58.8)	-	-
CD8+ cells frequency				0.001	10.190
	Low	28 (84.8)	07 (41.2)	-	-
	High	05 (15.2)	10 (58.8)	-	-

Data not reported: a01 for AC and 02 for LSCC b10 from AC and 6 from LSCC; c21 for AC and 11 for LSCC; d27 for AC and 6 for LSCC; e28 from AC and 6 from LSCC. Abbreviations: AC, actinic cheilitis; MD, moderately-differentiated; NA, not applicable; OSCC, oral squamous cell carcinoma; PD, poorly-differentiated; TILs, tumor-infiltrating lymphocytes and WD, well-differentiated. p-value < 0.05 considered statistically significant. χ² value: Chi-square test.

Histopathological analysis of AC lesions showed a predominance of intense solar elastosis (54.5%), mild inflammatory infiltrate (57.6%), mild dysplasia (54.6%) and presence of mitosis in 48.5% of cases. Intense inflammatory infiltrate was found only in male, with a significative association (χ²=7.337; p=0.026). There was a positive correlation between age and solar elastosis (ρ=0.341; p=0.042) and a negative correlation between inflammatory infiltrate and solar elastosis (ρ =-0.337; p=0.046).

Among LLSCC lesions, a higher prevalence of intense inflammatory infiltrate (41.2%), presence of mitosis (52.9%), well-differentiated tumors (52.9%), and TILs of up to 40% (52.9%) was found. A significant association between lesion group and inflammatory infiltrate (χ²=3.766; p=0.042) and a positive correlation between inflammatory infiltrate and TILs (ρ=0.521; p=0.032) were found.

### Immunoexpression analysis of PD-L1, CD4^+^ and CD8^+^ cells in AC and LLSCC and TILs in LLSCC lesions

**Figure 1:**
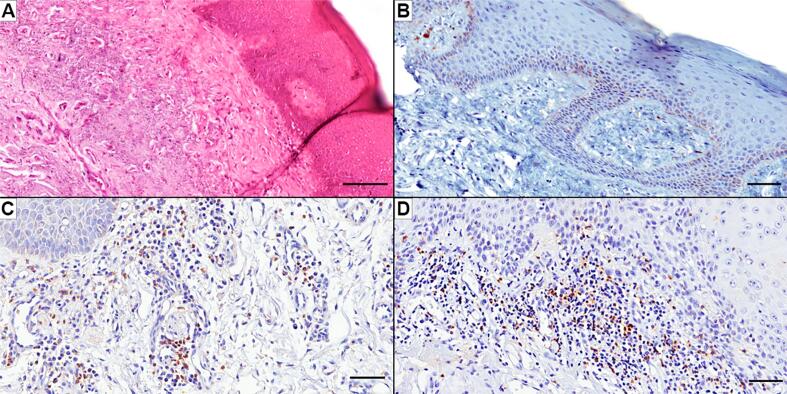
Histopathological and immunohistochemical analysis of a case of actinic chelitis. (A) Stained with hematoxylin and eosin, a case of actinic cheilitis that presented mild dysplasia, intense solar elastosis, and mild inflammatory infiltrate. (B): Immunoexpression of PD-L1 is shown mainly in dysplastic cells in the basal lane. (C) The case presented mild infiltration of CD4+ cells, mainly located in lamina propria. (D) Mild infiltration of CD8+ cells are also presented in the lamina propria. Scale bar of A: 100 μm. Scale bar of B, C, and D: 50 μm.

In relation to AC lesions, only six (18.2%) presented PD-L1 immunoexpression ranging from 1% (n=4) to 1% - 5% (n=2). In all PD-L1^+^ AC lesions (Figure 1A), the expression of this molecule was identified in the dysplastic cell membrane and the membrane and the cytoplasmic region of IC. As shown in Table 1, 72.7% of the cases had a low frequency of CD4^+^ cells and 84.8% had a low frequency of CD8^+^ cells. A positive correlation between dysplasia and CD8^+^ (ρ=0.308; p=0.049) was found. In most AC lesions, the CD4^+^ (Figure 1B) and CD8^+^ cells (Figure 1C) were distributed over the superficial portion of the lamina propria.

**Figure 2:**
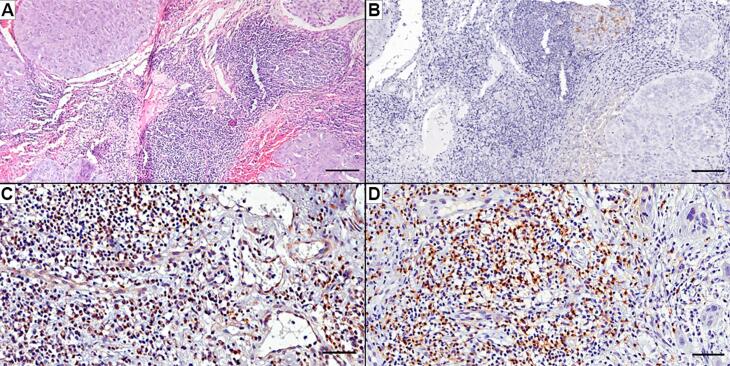
Histopathological and immunohistochemical analysis of a case of lower lip squamous cell carcinoma. (A): Stained with hematoxylin and eosin, a case of moderately differentiated lip carcinoma that presented a higher frequency of tumor-infiltrating lymphocytes, range between 41 to 90%. (B): Immunoexpression of PD-L1 is shown at the top of the image, mainly located in the membrane of tumor cells and some immune cells. (C): The case presented intense infiltration of CD4+ cells, which usually is seen in the tumor stroma. (D): Intense infiltration of CD8+ cells are also presented in tumor stroma. Scale bar of A and B: 100 μm. Scale bar of C and D: 50 μm.

Among the cases of LLSCC lesions, six (35.3%) showed PD-L1 immunoexpression ranging from 1% (n=3), 1% - 5% (n=2), and 5% - 20% (n=1). PD-L1 expression was identified in tumor cell membrane and in the membrane and cytoplasm of IC (Figure 2A). As presented in Table 1, the majority of cases (58.8%) had a high frequency of CD4^+^ cells and/or a high frequency of CD8^+^ cells. There was a positive correlation between the frequency of CD4^+^ and CD8^+^ cells (ρ=0.514; p=0.035), inflammatory infiltrate and CD4^+^ (ρ= 0.545; p=0.024), CD4^+^ and TILs (ρ=0.604; p=0.010), CD8^+^ and TILs (ρ=0.546; p=0.046), and PD-L1 and TILs (ρ=0.560; p=0.019). The CD4^+^ (Figure 2B) and CD8^+^ cells (Figure 2C), were distributed in the region of TILs and less frequently around the tumor.

Additionally, a higher frequency of CD4^+^ (χ² = 4.741; p=0.029), CD8^+^ (χ²=10.190; p=0.001), and PD-L1^+^ (χ²=3.822; p=0.046) was observed in the LLSCC compared to AC lesions.

In order to characterize the microenvironment of the AC (Table 2) and LLSCC lesions (Table 3), immunological parameters were dichotomized and correlated considering the groups formed based on the high or low frequency of CD4^+^ and CD8^+^ cells.

**Table 2:** Correlation between the frequency of CD4+, CD8+, histopathological characteristics, and PD-L1 immunoexpression in AC cases

Variables		CD4^low^CD8^low^	CD4^low^CD8^high^	CD4^high^CD8^high^	CD4^high^CD8^low^
Dysplasiaa (χ2 = 15.044; p < 0.05)					
	Mild	14	1	1	2
	Moderade	5	0	1	5
	Intense	2	2	0	0
Inflammatory infiltrateb (χ2 = 12.422; p < 0.05)					
	Mild	16	0	1	2
	Moderade	3	1	1	2
	Intense	2	2	0	3
Elastosis					
	Mild	4	0	1	0
	Moderade	6	1	0	3
	Intense	11	2	1	4
PD-L1c (χ2 = 8.942; p < 0.05)					
	Positive	4	2	0	0
	Negative	17	1	2	7
TOTAL		21	3	2	7

a dysplasia vs. CD4-CD8 groups;

b inflammatory infiltrate vs. CD4-CD8 groups;

c PD-L1 vs. CD4-CD8 groups.

Abbreviation: AC, actinic cheilitis; PD-L1, Programmed death ligand-1. p-value < 0.05 considered statistically significant. χ^2^-value: chi-square.

**Table 3:** Correlation between the frequency of CD4+, CD8+, histopathological characteristics, and PD-L1 immunoexpression in LLSCC cases

Variáveis		CD4^low^CD8^low^	CD4^low^CD8^high^	CD4^high^ CD8^high^	CD4^high^CD8^low^
Gradation					
	WD	2	0	5	2
	MD	2	2	1	0
	PD	1	0	2	0
Inflammatory Infiltrate					
	Mild	3	1	1	1
	Moderade	1	1	0	1
	Intense	1	1	3	0
TILsa (χ2 = 8.467; p < 0.05)					
	0% - 40%	5	2	4	1
	41% - 90%	0	1	5	2
PD-L1b (χ2 = 7.600; p < 0.05)					
	Positive	0	1	3	2
	Negative	5	1	5	0
TOTAL		5	2	8	2

a TILs vs. CD4-CD8 groups;

b PD-L1 vs. CD4-CD8 groups.

Abbreviations: LLSCC, lower lip squamous cell carcinoma; MD, moderately-differentiated; PD, poorly-differentiated; PD-L1, Programmed death ligand-1; TILs, Tumor infiltrate lymphocytes; WD, well differentiated. p-value < 0.05 considered statistically significant. χ^2^-value: chi-square.

In AC lesions, cases with low frequency of CD4^+^ and CD8^+^ were associated with the presence of mild dysplasia, mild inflammatory infiltrates, and intense solar elastosis. On the other hand, cases with a high frequency of CD4^+^ cells and/or a high frequency of CD8^+^ cells were associated with the presence of moderate to intense dysplasia, inflammatory infiltrate, and solar elastosis. All the cases with PD-L1 expression presented a low frequency of CD4^+^ cells. There was an association between dysplasia and CD4^+^-CD8^+^ groups (χ²=15.044; p=0.020), inflammatory infiltrate and CD4^+^-CD8^+^ groups (χ²=12.422; p=0.045), and PD-L1 expression and CD4^+^-CD8^+^ groups (χ²=8.942; p=0.047).

Cases of LLSCC with low frequency of CD4^+^ cells and/or low frequency of CD8^+^ cells were classified as moderately-differentiated tumors, with mild inflammatory infiltrate and low frequency of TILs. The majority of cases that presented PD-L1 expression had a high frequency of CD4^+^ and CD8^+^ cells. There was an association between TILs and CD4^+^-CD8^+^ groups (χ²=8.467; p=0.042) and PD-L1 expression and CD4^+^-CD8^+^ groups (χ²=7.600; p=0.046).

## Discussion

In Brazil, oral carcinoma is the fifth most common malignancy among men and the thirteenth among women.^25^ When it comes to lip carcinoma, the prognosis is usually better. However, the treatment has aggressive consequences, both functional and aesthetic.^26^ Malignant lesions of the lip are often preceded by PMOD, the epithelial changes of which occur due to high sun exposure. AC is the most representative lesion of this class.^27^ The importance of correct clinical-epidemiological characterization of AC is to prevent progression to labial squamous cell carcinoma.

The epidemiology of AC consists of the majoritarian of the lower lips in white men, above 40 years old, with an occupation related to sun exposure.^2^ These data corroborate those found in this study since both AC and LLSCC lesions were mostly identified among white men, aged over 50 years, some with occupations related to sun exposure, smokers, and alcoholics, 100% of AC lesions being on the lower lip.

Studies show that the association of sunlight exposure, socioeconomic factors, smoking, exposure to pesticides, diet, and genetic predisposition can accelerate the malignant transformation of AC.^28,29^ Most of the patients evaluated for the use of harmful habits in this study belong to the “unreported data” group. Thus, a limiting factor of this study is the method used to collect data from medical records and questionnaires, which are sometimes incomplete.^30^The absence of records such as occupation, smoking, and drinking significantly impairs the description of the epidemiological profile of the study.

Among the histopathological changes found in AC lesions, male individuals had a higher degree of inflammatory infiltrate, but this finding may be due to the higher proportion of male individuals included in this study. Furthermore, we found a positive correlation between age and solar elastosis, which can be explained by the accumulation of sun exposure.^2,31^

In this study, the negative correlation between inflammatory infiltrate vs. elastosis in lesions of patients with AC suggests that the degradation of the superficial dermis is directly related to the infiltration capacity of inflammatory cells in the connective tissue underlying the epithelium. Although there is no statistically significant correlation, most cases of AC were classified as mild inflammation, mild dysplasia, and intense elastosis. This data is reinforced by the association between dysplasia and CD4^+^/CD8^+^ groups, described in this study. From these data, it can be inferred that the presence of high-grade elastosis and the consequent less inflammatory juxtaepithelial infiltrate is associated with less architectural alteration of the epithelium, leading to mild dysplasia. These data suggest the importance of both inflammation and solar elastosis in the malignant transformation processes, proving the non-linearity of dysplasia in the evolution to carcinoma and the need for constant biopsies.^27,32^

Among the histopathological changes in LLSCC lesions, we prioritized the presence of inflammatory infiltrate, gradation of tumors, and the presence of TILs. Studies show the importance of the association of the density and distribution of TILs and the significant impact on the prognosis of patients with various types of carcinoma.^10,17^ However, few studies have analyzed this correlation in head and neck tumors and, mainly, in the oral cavity.^16,33^

In this study, we found a positive correlation between inflammatory infiltrate and TILs density, which indicates that recruited inflammatory cells still could infiltrate the tumor areas. Additionally, we found higher inflammatory infiltrate in LLSCC lesions when compared to AC. These results corroborate a previous study in melanocytic lesions^34^ and indicate that malignant cells are more immunogenic than dysplastic ones.

Cancer immunotherapy through the use of immune checkpoint inhibitors (anti-PD-1/anti-PD-L1) has gained prominence in recent years, since it results in increased survival of patients with different types of neoplasms.^7,8,11,13-15^ In oral malignancies, PD-L1 expression was associated with advanced stages of tumor^20,35^ and lymph node metastasis.^16,18,35,36^ Concomitant with the analysis of PD-1/PD-L1, it is known that the phenotypic and functional profile of the tumor microenvironment can determine mutual relations of carcinoma progression or regression and prognostic values of the patient; and these determining factors have already been evidenced in studies of oral malignant.^36-38^ Thus, the immunoexpression of PD-L1, in this study, was also related to the frequency of CD4^+^ and CD8^+^ cells in AC and LLSCC lesions and TILs in LLSCC lesions.

In the cases of AC, the expression of PD-L1 is predominant in the low frequency of CD4^+^ cells. This result suggests that morphological and genetic changes in dysplastic cells lead to an intrinsic induction of PD-L1 expression, similar to what happens in some carcinomas. Then, the expression of PD-L1 reduces proinflammatory signals and infiltration of inflammatory cells, such as CD4^+^ cells.^39^

On the other hand, in cases of LLSCC, most PD-L1^+^ had a high frequency of CD4^+^ and/or CD8^+^ cells, in the presence of a large number of TILs. The significant positive correlation between the frequency of CD4^+^/CD8^+^ cells and PD-L1 in AC and LLSCC lesions indicates that the CD4^+^/CD8^+^ ratio is different between the two functions and, therefore, the use of this parameter to differentiate groups, also as immunoexpression of PD-L1 are requested. Although the evaluation of factors that determine the evolution of AC to LLSCC is out of the scope of this study, it is known that the findings of inflammatory infiltrate and dysplastic changes of different degrees in the epithelium can determine the potential for the development of LLSCC.^40^

Data on the role of the PD-L1 molecule and the profile of the local immune response in certain types of PMOD, as well as in cases of LLSCC, are still scarce; however, the evidence points to different microenvironments between these types of lesions. Studies show that the tissue microenvironment and the soluble factors in malignant lesions – including oral squamous cell carcinoma (OSCC) and non-malignant diseases, such as AC, leukoplakia, and oral lichen planus – are significantly different in frequency and cell profile.^36-38,41^ While, in non-malignant diseases, there is a significant frequency of CD4^+^/PD-L1^+^ cells in the peripheral blood and less in lesions, in OSCC there is a higher frequency of CD4^+^/Foxp3^+^ cells restricted to the tumor microenvironment, with greater expression of PD-L1^+^, high levels of TGF-β and lower level of IFN-γ.^9,37^ This immunoregulatory profile via PD-1/PD-L1 interaction leads to the inhibition of effector T lymphocytes, a decrease in IFN-γ production, and proliferation of regulatory T cells; and it may be related to the evasion mechanisms of the immune response in OSCC.^37^

In addition, the expression of PD-L1 was associated with the inflammatory phenotype^33,42^ and, in this study, the presence of PD-L1 was more significant in carcinomas with a high frequency of CD8^+^ cells and a high density of TILs. Absent PD-L1 expression was predominant in tumors with a low frequency of CD8^+^ cells and a low density of TILs. It is known that CD8^+^ T lymphocytes and natural killer cells (NK) play an essential role in the antitumor immune response, through the production of IFN-γ and its cytotoxic activity.^43^ These mechanisms were associated with the types of PD-L1^+^/CD8^+^ carcinomas, with a correlation between the frequency of CD8^+^ T lymphocytes, their cytotoxic activity, and the PD-L1 molecule, that is, reflecting the adaptive immune response.^44^ Moreover, the overall survival rate of patients with this tumor profile is significantly more favorable compared to patients with a low frequency of CD8^+^ cells.^11^ Thus, it is necessary that when the total lymphocyte infiltrates is smaller and/or there is no balance between the infiltrates, the carcinomas tend to be less immunogenic, indicating that even non-specific, an inflammatory response can contribute to the recruitment of these cells to the site of the malignant neoplasm.^17^

In this study, we found that the highest expression of PD-L1 (up to 20%) were in lesions with a high frequency of CD8^+^. Although it was not possible to evaluate the functional profile of cells contained in the lesions in this study, it is suggested that the infiltration of CD8^+^ cells in LLSCC lesions are cytotoxic cells, responsible for the elimination of the altered antigens, thus following the morphological and cytological changes between the tissue.^9,37^

Most of the LLSCC cases in this study showed negative expression of PD-L1, which differs from studies published before.^36,38^ Thus, it is suggested that, in the patients included in this study, other unevaluated evasion pathways contributed to the progression of tumors, such as PD-L2, VISTA, and other molecules; in addition to mutations in cell cycle genes and immunosuppressive cytokines, inhibition of antigen processing, and presentation pathways and quantitative and functional decrease of immune cells.^45-47^

## Conclusion

Therefore, the PD-L1 molecule may be a potential escape route for the immune response in oral lesions, but the mechanisms differ between AC and LLSCC. In AC lesions included in this study, PD-L1 expression may occur through an intrinsic pathway, secondary to genetic and morphological changes in the epithelium. On the other hand, in the LLSCC included in this study, the expression of PD-L1 may be due to an adaptative mechanism, secondary to the production of IFN-γ by the cells of the immune system. We also highlight the importance of interpreting the microenvironment in a multifactorial context concomitant with the analysis of PD-L1 expression for a complete histological and cellular characterization. To improve the indication of immunotherapy in oral lesions, future experimental and clinical studies must be developed, considering the analysis of the inflammatory infiltrate, TILs, and other components of the immune microenvironment.
